# Hedgehog signal activation in oesophageal cancer patients undergoing neoadjuvant chemoradiotherapy

**DOI:** 10.1038/sj.bjc.6604361

**Published:** 2008-05-13

**Authors:** R Yoshikawa, Y Nakano, L Tao, K Koishi, T Matsumoto, M Sasako, T Tsujimura, T Hashimoto-Tamaoki, Y Fujiwara

**Affiliations:** 1 Department of Genetics, Hyogo College of Medicine, 1-1, Mukogawa-cho, Nishinomiya, Hyogo 663-8501, Japan; 2 Department of Surgery, Hyogo College of Medicine, 1-1, Mukogawa-cho, Nishinomiya, Hyogo 663-8501, Japan; 3 Institute for Advanced Medical Sciences, Hyogo College of Medicine, 1-1, Mukogawa-cho, Nishinomiya, Hyogo 663-8501, Japan; 4 Department of Pathology, Hyogo College of Medicine, 1-1, Mukogawa-cho, Nishinomiya, Hyogo 663-8501, Japan

**Keywords:** cancer stem cell, chemoradiotherapy (CRT), Gli-1, Hedgehog (Hh), oesophageal cancer

## Abstract

The zinc finger protein glioma-associated oncogene homologue 1 (Gli-1) is a critical component of the Hedgehog (Hh) signalling pathway, which is essential for morphogenesis and stem-cell renewal, and is dysregulated in many cancer types. As data were not available on the role of Gli-1 expression in oesophageal cancer progression, we analysed whether it could be used to predict disease progression and prognosis in oesophageal cancer patients undergoing neoadjuvant chemoradiotherapy (CRT). Among 69 patients with histologically confirmed oesophageal squamous cell carcinomas (ESCCs), 25 showed a pathological complete response after preoperative CRT. Overall survival (OS) was significantly associated with lymph-node metastasis, distant metastasis, and CRT, and was further correlated with the absence of both Gli-1 nuclear expression and residual tumour. All patients with Gli-1 nuclear expression (10.1%) had distant or lymph-node metastasis, and six out of seven died within 13 months. Furthermore, patients with Gli-1 nuclear-positive cancers showed significantly poorer prognoses than those without (disease-free survival: mean DFS time 250 *vs* 1738 months, 2-year DFS 0 *vs* 54.9%, *P*=0.009; OS: mean OS time 386 *vs* 1742 months, 2-year OS 16.7 *vs* 54.9%, *P*=0.001). Our study provides the first evidence that Gli-1 nuclear expression is a strong and independent predictor of early relapse and poor prognosis in ESCC after CRT. These findings suggest that Hh signal activation might promote cancer regrowth and progression after CRT.

The Hedgehog (Hh) signalling pathway plays an important role in cell growth and differentiation, with normal roles in embryonic pattern formation and adult tissue homoeostasis, and has a pathological function in tumour initiation and growth including foregut development. In addition, it regulates the response of adult stem cells during the regeneration that takes place after tissue damage (Ingham and McMahon, 2001; van den Brink *et al*, 2001; Wetmore, 2003; Beachy *et al*, 2004). Secreted Hh molecules bind to the predominant Hh receptor patched1 (Ptch1), alleviating the Ptch-mediated suppression of smoothened (Smo), which is a putative seven-transmembrane protein, and triggering a cascade of intracellular events including the nuclear translocation of the zinc finger protein glioma-associated oncogene homologue 1 (*Gli-1*; Nakano *et al*, 1989; Aza-Blanc *et al*, 1997; Ruiz i Altaba, 1999; Hooper and Scott, 2005).

Mutational activation of the Hh signalling pathway, as in Gorlin's syndrome, is associated with tumorigenesis in a small subset of tissues, predominantly the skin, cerebellum, and skeletal muscle (Johnson *et al*, 1996). It is also implicated in carcinogenesis of the gastrointestinal tract as well as basal cell carcinoma, medulloblastoma, and prostate, pancreatic, and breast cancers. The importance of Hh signal activation has been demonstrated by the ability of Hh pathway antagonists to suppress the growth of gastrointestinal tumour cell lines and xenografts (Berman *et al*, 2003; Ma *et al*, 2005; Ohta *et al*, 2005).

Oesophageal cancer is virulent, and the presence of lymph-node metastasis and vascular invasion indicate a high malignant potential (Parkin *et al*, 2005). Surgery is the treatment of choice for patients with locoregionally confined oesophageal cancer (stages II and III); however, the 5-year survival rate is less than 20%, even after curative surgery (Parkin *et al*, 2005). Since 1996, we have combined preoperative chemoradiotherapy (CRT) with radical surgery for the treatment of oesophageal cancers, and have reported increased resectability, a reduced incidence of both local recurrence and distant metastasis, and a better prognosis for CRT responders (Fujiwara *et al*, 2005). However, the benefits of CRT are controversial, and some clinical trials have shown that this preoperative strategy benefits only the 25% of patients who show a pathological complete response (CR; that is, no cancer cells in the resected specimen), whereas the remaining 75% present CRT-resistant and highly aggressive cancers with lymph-node and distant metastases (Coia *et al*, 2000; Iyer *et al*, 2004). Mortality from this disease remains high because current therapies are limited by the emergence of therapy-resistant cancer cells.

Recent evidence has suggested a previously uncharacterised model of solid tumour biology with a defined subset of tumour cells harbouring stem cell-like properties of self-renewal. This tumorigenic population of cancer cells is also characterised by differentiation, heterogeneity, and the ability to maintain tumour growth while playing an important role in both tumorigenesis and resistance to therapeutics (Al-Hajj *et al*, 2003; Dick, 2003). However, research has been limited because of the lack of distinct molecular makers on these cancer progenitor cells. Aberrant Hh pathway activation might be involved in the maintenance of such a population in cancer, because Hh signalling regulates progenitor-cell fate in normal development and homoeostasis.

There are no reported data on the role of Gli-1 expression, which is a key Hh pathway target, in oesophageal cancer progression after CRT. Moreover, the prognosis of patients undergoing CRT has not yet been reliably estimated. The current study retrospectively investigated the expression of Gli-1 protein in human oesophageal squamous cell carcinoma (ESCC) tissues, and evaluated the clinical implications of Hh signal activation for these patients who underwent preoperative CRT and radical surgery.

## PATIENTS AND METHODS

### Patients and therapy

**Figure 1:**
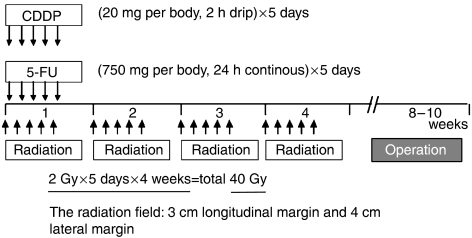
Schedule of preoperative CRT.

In total, 69 patients (11 women and 58 men; mean age, 60.7 years; age range, 38–78 years) with surgically excised ESCCs were studied at the Hyogo College of Medicine, Japan, between April 1996 and December 2005. For preoperative CRT, 5-flurouracil (5-FU; 500 mg m^−2^ per day) was administered as a 120-h continuous intravenous (i.v.) infusion starting on day 1, and cisplatin (CDDP; 15 mg m^−2^ per day) was administered as a 2-h i.v. infusion on days 1–5 (Figure 1). Radiation therapy was performed after CDDP infusion on days 1–5 using a linear accelerator (Mevatron KD2; Siemens, Munich, Germany) and a previously described radiation method (Fujiwara *et al*, 2005). Chemotherapy was combined with radiation therapy during the first week, and then radiation therapy alone was repeated for the next 3 weeks (days 8–12, 15–19, and 22–26). Each single dose of radiation was 2 Gy per day, giving a total dose of 40 Gy. Surgery was usually performed 4–6 weeks after the completion of CRT. Follow-up information was obtained from office charts, hospital records, and telephone interviews. The ethics committee of the institution approved the study protocol.

### Evaluation prior to surgery

Approximately 2–4 weeks after the completion of CRT, patients underwent a complete staging workup according to Response Evaluation Criteria in Solid Tumours guidelines. Patients were considered to have clinical CR to CRT if no residual tumour was detected at endoscopy and if no occurrence of metastatic disease was identified on computed tomography scan evaluation, and to have partial response if at least a 30% decrease in the sum of the longest diameter of target lesions.

### Immunohistochemistry

Oesophageal squamous cell carcinoma tissue specimens were cut open longitudinally, fixed with formalin, and then processed using conventional procedures. Sections were heated for 20 min at 98°C in 10 mmol l^−1^ sodium citrate (pH 6.0) for antigen retrieval. They were then incubated with goat polyclonal antibody against human Gli-1 (C-18; Santa Cruz Biotechnology, Santa Cruz, CA, USA) and visualised with the Envision kit (Dako Cytomation, Glostrup, Denmark). Alternative primary antibodies included goat polyclonal anti-human cyclooxygenase (COX)-2 antibody, mouse monoclonal anti-human vascular endothelial growth factor (VEGF) antibody (Santa Cruz Biotechnology), and mouse monoclonal anti-human chemokine (C-X-C motif) receptor 4 (CXCR4) antibody (R&D systems, Minneapolis, MN, USA). Slides were developed with 3,3′-diaminobenzidine tetrahydrochloride solution containing 1 ml l^−1^ H_2_O_2_, lightly counterstained with haematoxylin, and then mounted. Normal mouse immunoglobulin G (IgG) was substituted for the primary antibody as a negative control. Sections were examined microscopically by two of the authors (LT and TT) without knowledge of clinicopathological features. The results of nuclear expression were graded on a five-point scale based on the percentage of specific tumour-cell staining as follows: no specific staining (−); <5% of the tumour cells (±); ⩾5 to <35% of the tumour cells (+); ⩾35 to <65% of the tumour cells (++); and ⩾65% tumour cells (+++). Subtle expression was defined as <5% of the tumour cells (±).

### Statistical analysis

Overall survival (OS) was defined as the time from the initial diagnosis to either the patient's death or the date of the last available information on vital status. Disease-free survival (DFS) was defined as the length of time after treatment during which no cancer was found. In univariate analysis, the difference between the cumulative survival rates of the patient groups was calculated by the log-rank test for comparison using Kaplan–Meier survival curves. Statistical significance was considered at values of *P*<0.05. Statistical analyses were carried out using STATISTICA statistical software, version 06J (STATISTICA, Tulsa, OK, USA).

## RESULTS

### Patient and tumour characteristics

The patient characteristics are summarised in Table 1. The patient gender bias was male (male/female=58 : 11). The tumour histology was ESCC in all cases, with 67 tumours (97.1%) originating in the thorax. According to the tumour-node metastasis system of the American Joint Committee on Cancer, stage II tumours were seen in 29 patients (42.0%), stage III tumours were seen in 26 patients (37.7%), and stage IV tumours were seen in 14 patients (20.3%). In total, 29 patients (42.0%) had lymph-node metastasis at the time of diagnosis. All lesions before CRT presented with a T_3_ or T_4_ extent of invasion. Three-quarters of the patients had tumours that were between 6 and 8 cm in diameter. An M+ classification was described in 14 tumours (M_1a_ in six patients and M_1b_ in eight patients). Seven patients had distant metastasis of the liver. All patients experienced a disease-free period. During the follow-up period, 10 patients (14.5%) developed local recurrence or residual tumours, eight patients (11.6%) developed neck or coeliac lymph-node recurrence, and 14 patients (20.3%) developed distant metastasis. In total, 33 patients (47.8%) died during follow-up; among these, 29 patients (42.0%) died from their tumours, whereas the remaining five patients (7.2%) were tumour-free and died of intercurrent diseases.

## GLI-1 NUCLEAR EXPRESSION PREDICTS EARLY RECURRENCE AND POOR PROGNOSIS

**Figure 2:**
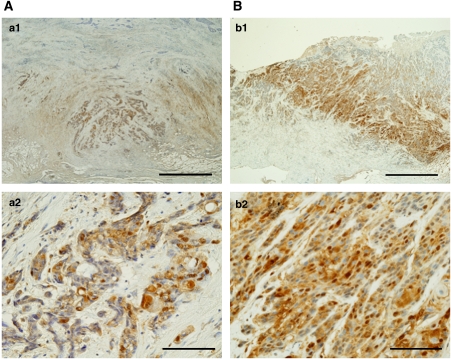
Immunohistochemical detection of Gli-1 in ESCC specimens. (**A**) Subtle nuclear expression for Gli-1 (**a1**, × 20; **a2**, × 200). (**B**) Strong nuclear expression for Gli-1 (**b1**, × 20; **b2**, × 200). Bars indicate 1 mm ( × 20) or 100 *μ*m ( × 200).

Twenty-five tumours were totally eradicated by CRT, resulting in an absence of visible tumour cells: a pathological complete CR. Gli-1 expression was absent in 12 tumours (17.4%) but present in 31 tumours (44.9%), seven (10.1%) of which were nuclear and 26 (37.7%) of which were cytoplasmic. In the tumours with Gli-1 nuclear expression, two were scored as (±), two were scored as (+), three were scored as (++), and none were scored as (+++) (Figure 2). All seven Gli-1 nuclear-positive cancers expressed CXCR4, COX-2, and VEGF. In total, 14 out of 69 patients (20.3%) showed positive expressions of all three markers. All patients with Gli-1 nuclear expression had distant or lymph-node metastasis, and six of the seven died within 13 months. Even the 3.0% of patients who were scored as (±) died of lung or neck lymph-node metastasis within 10 months of surgery. In the Gli-1 nuclear expression group, recurrences were found in all seven patients; these were local in three cases and distant (in the liver, bone, lung, and neck lymph nodes) in four cases. In contrast, in the Gli-1-null or cytoplasmic expression or no residual tumour group (*n*=62), recurrences were found in 22 patients (in the liver, bone, lung, neck lymph nodes, and thyroid gland). Statistical analysis showed that distant recurrence was more common in the patients with Gli-1 nuclear expression (*P*=0.002, hazard ratio 4.115, 95% confidence interval (CI) 1.676–10.104). The univariate analysis of the OS prognostic factors is summarised in Table 2. Lymph-node metastasis and distant metastasis had a significant prognostic value (*P*=0.0001, *P*=0.0001, respectively). Furthermore, patients showing effect of CRT revealed significantly better OS prognosis compared to those without it (*P*=0.0001).

**Figure 3:**
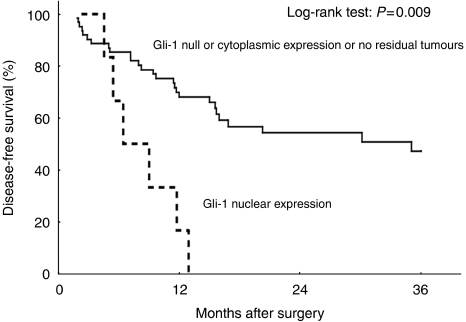
Disease-free survival differences between patients with nuclear Gli-1 expression and patients with no residual tumour and Gli-1-null or cytoplasmic expression.

**Figure 4:**
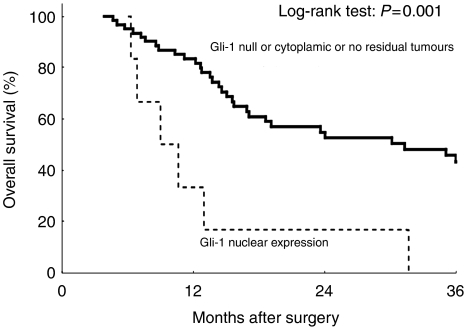
Overall survival differences between patients with Gli-1 nuclear expression and patients with no residual tumour and Gli-1-null or cytoplasmic expression.

Kaplan–Meier analysis suggested that the prognosis was particularly unfavourable for patients with Gli-1 nuclear expression in their primary tumours, compared with the Gli-1-null or cytoplasmic expression patients or those with no residual tumour. The mean DFS time of the Gli-1 nuclear expression group was 250.8±38.7 months (95% CI 174.9–326.8 months) compared with 1734.8±212.2 months (95% CI 1318.8–2150.8 months) for the Gli-1-null or cytoplasmic expression or no residual tumour group (Figure 3; *P*=0.009); the mean OS times for these groups were 386.7±116.6 months (95% CI 158.1–615.3 months) and 1742.7±208.5 months (95% CI 1334.2–2151.3 months), respectively (Figure 4; *P*=0.001). The 1-year, 2-year, and 3-year DFS rates for the Gli-1 nuclear expression group *vs* the Gli-1-null or cytoplasmic expression or no residual tumour group were 16.7 *vs* 68.9%, 0 *vs* 54.9%, and 0 *vs* 47.8%, respectively; the corresponding 1-year, 2-year, and 3-year OS rates were 33.3 *vs* 82.8%, 16.7 *vs* 54.9%, and 0 *vs* 46.1%.

## DISCUSSION

Our previous studies showed that persistent positive CXCR4, COX-2, or VEGF expression could predict earlier recurrence in ESCC patients after CRT. Furthermore, CXCR4 expression could predict shorter OS period (Koishi *et al*, 2006; Yoshikawa *et al*, 2007). These findings suggested that metastatic behaviour was determined, at least in part, by chemokine networks or microvasculature on the surface of tumour cells in response to external therapeutic intervention (ie, CRT). Intriguingly, our data showed that Gli-1 nuclear expression, Hh signal activation, as well as CXCR4 expression were more important than COX-2 or VEGF expressions in tumour relapse and prognosis after CRT, whereas appropriate postoperative chemotherapy could improve the OS period even after the disease progression associated with COX-2 or VEGF expression. Gli-1 nuclear expression after CRT might therefore be a useful and reliable biomarker for the screening and management of high-risk patients with poorer prognosis. To date, this is the first report to demonstrate the prognostic importance of Hh signal activation in ESCC patients undergoing CRT, the application of Gli-1 analysis to all post-CRT ESCC patients, and its utilisation in the planning of therapeutic strategies. In some cases, supportive care emphasising quality of life rather than aggressive anti-cancer therapy might be recommended to high-risk patients with Hh signal activation.

Increased understanding of the importance of morphogens as stem-cell markers allows a more targeted approach to therapy for individual tumours. In normal tissues, Hh-induced progenitor-cell proliferation is transient and tightly regulated, resulting in acute epithelial repair and regeneration after injury (Beachy *et al*, 2004). In contrast, aberrant activation of tumour Hh signalling cascades is not controlled by regulatory mechanisms, leading to support survival of tumour clonogens and tumour regrowth after CRT in an autocrine–paracrine manner. These tumorigenic populations of ‘more aggressive’ cancer cells that can initiate relapse and maintain disease are explained by the ‘cancer stem cell’ concept, but not by the stochastic carcinogenesis model, in which cells from a heterogeneous tumour are each able to randomly give rise to new cancers. In the hierarchical cancer stem cell model, only a small group of undifferentiated or poorly differentiated cells, representing less than 5% of the total tumour mass, can repopulate. The proof of this concept for cancer-initiating cells has been established in various malignancies including acute myelogenous leukaemia, and breast and brain cancers (Lapidot, 1994; Al-Hajj *et al*, 2003; Singh *et al*, 2004).

In the current study, even patients with Gli-1 nuclear (±) expressing cancers (2.9%) died from distant metastasis within a few months. Our results also suggest that Gli-1 activation is more important than the abovementioned parameters in orientating tumour biology and prognosis after CRT. Indeed, postoperative adjuvant chemotherapy failed to improve the prognosis of patients with Gli-1 nuclear-positive tumours. Any therapeutic intervention could not arrest the disease progression in association with Hh signal activation. These results suggest that Hh signal activation might promote cancer regrowth and progression after CRT, and that subtle Gli-1 activation after CRT is indicative of the emergence of ‘more aggressive’ cancer cells. The functional properties of such cells might be influenced through external signals, including those that can affect migratory potential and are mediated by further-differentiated cancer cells and host stromal cells. The signals that can influence migratory potential are thought to result in highly aggressive cancer forms, and thereby contribute to cancer recurrence. In this paper, the effect of CRT on Gli-1 expression was not revealed. A recent report has shown that Gli-1 was expressed in a half of ESCC patients’ specimens (Mori *et al*, 2006). Our results suggested that Gli-1 expression with CRT was less than that without it (10.1 *vs* 50.0%), and that Hh activation even after CRT correlated with earlier relapse and treatment resistance. As expected, all Gli-1 nuclear-positive cancers in the current study expressed CXCR4, COX-2, and VEGF, which was indicative of a cancer progenitor cell-like surface characteristic that might contribute to cancer relapse and treatment failure.

Our current study focused on the novel therapeutic targeting of distinct oncogenic signalling elements activated in cancer progenitor cells and their local microenvironment. Recently, a combination of gemcitabine and small-molecule *Smo* inhibitor cyclopamine has been shown to abrogate metastases in pancreatic cancer xenografts, whereas gemcitabine alone reduced the size of primary ‘bulk’ tumours (Feldmann *et al*, 2007). The efficacious blockade of Hh cascades activated in cancer progenitor cells during tumour progression shows promise for improving current clinical treatments against high-risk, metastatic, or relapsed ESCC. Future clinical application of Hh pathway antagonists such as cyclopamine and arsenic trioxide should be explored.

In summary, we showed that the monoclonal amplification of Gli-1 nuclear localisation implicating Hh pathway activation was correlated with oesophageal cancer progression, and that subtle Hh cascade activation after CRT indicated early cancer progenitor cell emergence. Although the functional and clinical significance of the Hh pathway remains to be elucidated, we suggest that Gli-1 expression is a link between relapse and CRT-resistant cancer cells, and is thus a potential diagnostic biomarker and therapeutic target.

## Figures and Tables

**Table 1:** Patient characteristics

**Characteristics**	***n***
Sex (male/female)	69 (58/11)
Mean age, years (range)	60.7 (38–76)
	
*Location of tumour*	
Cervix	2
Upper thorax	8
Middle thorax	39
Lower thorax	20
	
*T-classification*	
T_3_	40
T_4_	29
	
*N-classification*	
N_0_	40
N_1_	29
	
*M-classification*	
M_0_	55
M_1_	14
	
*UICC TNM stage*	
IIa	29
III	26
IVa	7
IVb	7

TNM=tumour-node metastasis; UICC=International Union Against Cancer.

**Table 2:** Univariate analysis of prognostic factors for OS

**Covariate**	***n***	**Hazard ratio**	**95% CI**	***P*-value for OS**
*Age (years)*				
<70	54	0.984	0.448–2.160	0.968
⩾70	15			
				
*Gender*				
Male	58	0.456	0.161–1.291	0.139
Female	11			
				
*Effect of CRT*				
Effective	49	0.207	0.104–0.413	0.0001^***^
Not effective	20			
				
*Lymph-node metastasis*				
Positive	31	3.567	1.751–7.266	0.0001^***^
Negative	38			
				
*Distant metastasis*				
Positive	13	4.064	2.016–8.192	0.0001^***^
Negative	56			
				
*Depth of tumour invasion*				
T_3_	40	0.383	0.195–0.751	0.005^*^
T_4_	29			
				
*Tumour location* a				
Upper	10	1.139	0.442–2.934	0.787
Lower	59			
				
*Gli-1 expression*				
Nuclear	7	4.115	1.676–10.104	0.002^**^
Others	62			

CI=confidence interval; CRT=chemoradiotherapy; Gli-1=glioma-associated oncogene homologue 1; OS=overall survival.

^*^P<0.01; ^**^P<0.005; ^***^P<0.0005.

a Upper or lower, above or below the tracheal bifurcation.
